# Comparison of Venous Thromboembolism Outcomes after COVID-19 and Influenza Vaccinations

**DOI:** 10.1055/a-2183-5269

**Published:** 2023-12-04

**Authors:** Manila Gaddh, David Scott, Waldemar E. Wysokinski, Robert D. McBane, Ana I. Casanegra, Lisa Baumann Kreuziger, Damon E. Houghton

**Affiliations:** 1 Department of Hematology and Medical Oncology, Emory University School of Medicine, Atlanta, Georgia, United States; 2 Department of Hematology and Medical Oncology, Medical College of Wisconsin, Milwaukee, Wisconsin, United States; 3 Versiti, Blood Research Institute, Milwaukee, Wisconsin, United States; 4 Division of Vascular Medicine, Department of Cardiovascular Diseases, Mayo Clinic, Rochester, Minnesota, United States; 5 Division of Hematology, Department of Medicine, Mayo Clinic, Rochester, Minnesota, United States

**Keywords:** COVID-19 vaccination, venous thromboembolism, thrombosis

## Abstract

**Background** Published data on the risk of venous thromboembolism (VTE) with coronavirus disease 2019 (COVID-19) vaccines are scarce and inconclusive, leading to an unmet need for further studies.

**Methods** A retrospective, multicentered study of adult patients vaccinated for one of the three approved COVID-19 vaccines in the United States of America and a pre-COVID-19 cohort of patients vaccinated for influenza at two institutions: Mayo Clinic Enterprise sites and the Medical College of Wisconsin, looking at rate of VTE over 90 days. VTE was identified by applying validated natural language processing algorithms to relevant imaging studies. Kaplan–Meier curves were used to evaluate rate of VTE and Cox proportional hazard models for incident VTE after vaccinations. Sensitivity analyses were performed for age, sex, outpatient versus inpatient status, and type of COVID-19 vaccine.

**Results** A total of 911,381 study subjects received COVID-19 vaccine (mean age: 56.8 [standard deviation, SD: 18.3] years, 55.3% females) and 442,612 received influenza vaccine (mean age: 56.5 [SD: 18.3] years, 58.7% females). VTE occurred within 90 days in 1,498 (0.11%) of the total 1,353,993 vaccinations: 882 (0.10%) in the COVID-19 and 616 (0.14%) in the influenza vaccination cohort. After adjusting for confounding variables, there was no difference in VTE event rate after COVID-19 vaccination compared with influenza vaccination (adjusted hazard ratio: 0.95 [95% confidence interval: 0.85–1.05]). No significant difference in VTE rates was observed between the two cohorts on sensitivity analyses.

**Conclusion** In this large cohort of COVID-19-vaccinated patients, risk of VTE at 90 days was low and no different than a pre-COVID-19 cohort of influenza-vaccinated patients.

## Introduction

Coronavirus disease (COVID-19), caused by the severe acute respiratory syndrome coronavirus 2, spread worldwide within a short period of time leading to a pandemic that infected millions of people. 1 Higher risk of venous thromboembolism (VTE) with COVID-19 infection was recognized early in the pandemic and has varied with virus strain. 2 3 Comparative data from patients hospitalized with COVID-19 versus those hospitalized with influenza have shown a higher rate of VTE in the former cohort suggesting a distinct thrombogenicity associated with COVID-19 virus. 4 Vaccination against COVID-19 reduced the risk of hospitalization and death 1 5 but raised concerns about complications including the risk of VTE after COVID-19 vaccination. 6

A rare thrombotic complication, termed vaccine-induced immune thrombotic thrombocytopenia (VITT), with two adenovirus vectored vaccines, CHaDOx1 nCov-19 AstraZeneca and AD26.COV2.S Johnson & Johnson, was recognized in early 2021. 7 8 9 Unlike typical VTE episodes which tend to occur in the extremities and lungs, VITT was associated with thrombosis in unusual sites such as visceral locations and the cerebral venous sinuses. 10 VITT also follows a distinct pathophysiology related to production of antibodies to platelet factor-4. 11 After a temporary pause in the vaccination program for the two vaccines associated with VITT cases, both the Center for Disease Control and Prevention, United States of America and World Health Organization advised resumption of their use based on an in-depth review which showed that the events were rare and the risk–benefit ratio remained favorable overall. The concern about this specific thrombotic concern, which was well publicized, contributed to increased vaccine hesitancy and prompted the investigation of thrombotic events more generally with the vaccines.

Recent data from two states in the United States linking excess mortality with low vaccination rates despite widespread availability of COVID-19 vaccines highlight the need for data on safety of COVID-19 vaccines 12 ; such data may in turn help address vaccine hesitancy. Published data on the risk of VTE following various COVID-19 vaccines are limited and inconclusive; this includes a single-center study from the United States and a few international studies looking at specific COVID-19 vaccines and/or populations, which precludes generalizability of the results. 13 14 15 16 While most studies have shown no significant increased risk with COVID-19 vaccines, one large study from Argentina did find a higher thrombotic risk when comparing COVID-19 to influenza vaccines. 17

To better evaluate COVID-19 vaccination risk for venous thromboembolic events, we conducted this real-world observational study comparing rates of VTE after vaccination with the three U.S. Food and Drug Administration (FDA)-approved COVID-19 vaccines with VTE rates after a pre-COVID-19 cohort of patients who received influenza vaccinations.

## Methods

### Study Design

A retrospective cohort study of patients vaccinated for COVID-19 and influenza at two institutions: the Mayo Clinic Enterprise sites (including Rochester, Minnesota, Jacksonville, Florida, Scottsdale Arizona, and Mayo Clinic Health System sites) and the Medical College of Wisconsin. The study was approved by institutional review boards at both institutions.

### Patient Population

Adult patients 18 years of age or older who were vaccinated for COVID-19 (Pfizer-BioNTech *mRNA vaccine* , Moderna mRNA vaccine, or Janssen/Johnson & Johnson adenovirus vaccine) between November 1, 2020 and November 1, 2021 and influenza-vaccinated patients between July 1, 2019 and April 1, 2020 were identified via electronic health records from the two institutions.

### Covariates

Baseline demographic data including age (continuous), sex (male/female), and race (white/non-white) were extracted from the medical records. Baseline comorbidities were extracted using International Classification of Diseases, Tenth Edition (ICD-10) codes and included if present prior to the date of vaccination (first vaccination for Moderna and Pfizer vaccines). Comorbidities from the Charlson comorbidity index (CCI) (acute myocardial infarction, cancer, cerebrovascular accident, congestive heart failure, connective tissue disease, dementia, diabetes, human immunodeficiency virus, liver disease, hemiplegia, peptic ulcer disease, pulmonary disease, peripheral vascular disease, and renal disease) as well as atrial fibrillation, hypertension, hyperlipidemia, and history of VTE were extracted using ICD-10 codes. The outpatient versus inpatient status of the patients at the time of vaccination was recorded from the encounter type or inpatient medication-administration reconciliation record for each patient. COVID-19 infection was not included as a covariate as the influenza-vaccinated cohort occurred prior to the COVID-19 pandemic and patients with vaccination during this time frame could not have been affected.

### Study Outcomes

The aim of this study was to compare the rates of VTE occurring within 90 days after influenza and COVID-19 vaccinations. Since previously vaccinated influenza patients with a history of VTE could have an ICD-10 code for VTE used in follow-up visit (possibly after COVID-19 vaccination), our primary outcome was imaging confirmed acute VTE (upper or lower extremity deep vein thrombosis [DVT] or pulmonary embolism [PE]) occurring within 90 days after the vaccination. To identify acute VTE, all imaging studies that could diagnose VTE (computed tomography chest studies with intravenous contrast, upper or lower extremity Duplex ultrasound) were extracted and the radiology report text was analyzed using validated natural language processing (NLP) algorithms at each institution. 18 19 The NLP algorithms were specifically validated to distinguish between acute and nonacute VTE.

### Statistical Analysis

Baseline characteristics were compared between patients vaccinated for COVID-19 or influenza. Categorical variables were compared examining number and percentages and analyzed using Pearson's Chi-squared test. Continuous variables were examined using the mean and standard deviation (SD) and analyzed using Student's *t* -test. The date of the first influenza and COVID-19 vaccination during the study period was considered the index date and VTE events occurring after this date were analyzed using time elapsed from the index date. VTE events occurring on the day of vaccination were included in the frequency of VTE events but were not included in the time-to-event analyses. Kaplan–Meier curves were made examining the rate of VTE between vaccination groups. Cox proportional hazard models were used to calculate adjusted and unadjusted hazard ratio (aHR and uHR) comparing vaccine type for incident VTE events occurring within 90 days. aHR was calculated using CCI (without age), age, sex, race, atrial fibrillation, hypertension, hyperlipidemia, history of VTE, and outpatient versus inpatient status of patients at the time of vaccination. Additional stratified-sensitivity analyses were performed for age (<65 years vs. ≥ 65 years), sex, outpatient versus inpatient status of patients, and type of COVID-19 vaccine.

## Results

A total of 911,381 patients were identified who received one of the three U.S. FDA-approved COVID-19 vaccines between November 1, 2020 and November 1, 2021, and 442,612 patients who received influenza vaccine between July 1, 2019 and April 1, 2020. The most common COVID-19 vaccine received was Pfizer (523,233), followed by Moderna (332,784) then Janssen (55,364). A total of 489,872 (93.62%) patients received a second dose of Pfizer vaccine and 307,236 (92.32%) patients received a second dose of Moderna vaccine at a median of 21 days (range: 17–347 days) and 28 days (range: 19–343 days) after the first dose, respectively.

Table 1 shows the baseline characteristics of the study population. Overall, most of the study population were from the Mayo Clinic Enterprise sites (84.64%). The mean age was 56.8 years (SD: 18.3) and 56.5 years (SD: 18.3), and 55.32 and 58.65% were females in the COVID-19-vaccinated and influenza-vaccinated cohorts, respectively. Most patients (86.59% in the COVID-19 vaccination cohort and 90.41% in the influenza vaccination cohort) were white. The influenza vaccination cohort had a higher mean CCI and higher prevalence of other comorbidities including atrial fibrillation, hyperlipidemia, hypertension, and history of VTE, as compared with the COVID-19 vaccination cohort. Distribution of all comorbidities included in the CCI among the two cohorts are shown in Supplementary Table S1 (online only).

**Table 1:** Baseline characteristics

Characteristic	Covid-19 vaccinated, *N* = 911,381	Influenza vaccinated, *N* = 442,612	*p-* Value
Age, mean (SD)	56.8 (18.3)	56.5 (18.3)	<0.001
Sex, female, *n* (%)	504,072 (55.32)	259,597 (58.65)	<0.001
Race, white, *n* (%)	788,451 (86.59)	399,855 (90.41)	<0.001
CCI, mean (SD)	1.35 (2.19)	1.57 (2.36)	<0.001
Other comorbidities, *n* (%)			
Atrial fibrillation	68,263 (7.49)	46,049 (10.40)	<0.001
Hyperlipidemia	301,709 (33.10)	204,778 (46.27)	<0.001
Hypertension	286,147 (31.40)	187,742 (42.42)	<0.001
History of VTE	32,463 (3.56)	22,126 (5.00)	<0.001
Site, *n* (%)			
Medical College of Wisconsin	119,371 (13.10)	88,573 (20.01)	
Mayo Clinic Enterprise	792,010 (86.90)	354,039 (79.99)	

Abbreviations: CCI, Charlson comorbidity index; SD, standard deviation; VTE, venous thromboembolism.

### Primary Outcome

VTE occurred within 90 days in 1,498 (0.11%) of the total 1,353,993 vaccinations, including 882 (0.10%) in the COVID-19 vaccination cohort and 616 (0.14%) in the influenza vaccination cohort. The rates of VTE in the three different types of COVID-19 vaccine cohorts were similar, specifically 0.09% with Pfizer, 0.11% with Moderna, and 0.08% with Janssen.

**Fig. 1:**
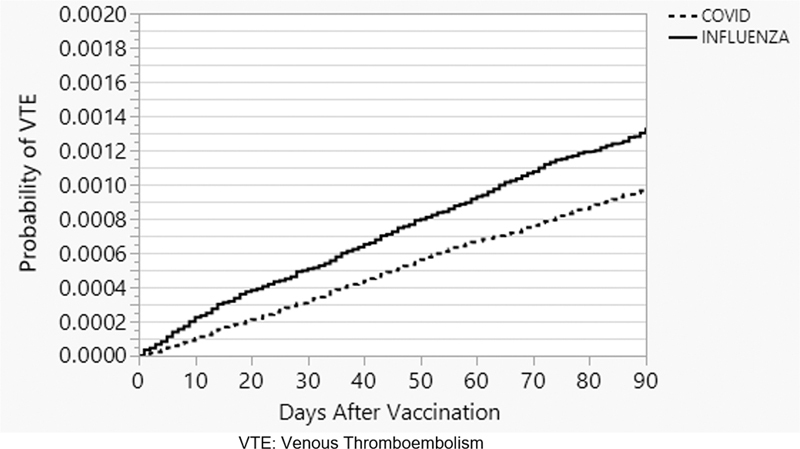
Kaplan–Meier curves of venous thromboembolism.

Fig. 1 shows the time to VTE curves in the COVID-19 and influenza vaccination cohorts. In total, 30 patients (2 COVID-19 and 28 influenza) were excluded from time to event analyses as their VTE event occurred on the day of their vaccination and could not definitively be classified as having occurred before or after the injection. The uHR for VTE overall for COVID-19 compared with influenza vaccination was 0.72 (95% confidence interval [CI]: 0.65–0.80), indicating a lower rate of VTE in the COVID-19-vaccinated cohort. After multivariable adjustment for age, sex, race, comorbidities, and outpatient versus inpatient status of patients, no difference in rates was observed, aHR was 0.95 (95% CI: 0.85–1.05).

### Sensitivity Analyses

Additional sensitivity analyses were performed to understand the effect of specific variables on the rates of VTE seen in the two vaccination cohorts. The aHR for VTE with COVID-19 vaccination after stratification for age (<65 years vs. ≥ 65 years), sex, outpatient versus inpatient status of patients, and type of COVID-19 vaccine (mRNA vs. adenoviral vector) is summarized in Table 2 . Interestingly, the aHR was slightly higher in the younger age group of <65 years, females, inpatients, and with adenoviral-vector vaccine as compared with their respective comparative stratum, but no significant difference in VTE rates was observed between COVID-19 and influenza vaccination cohorts in any of the stratified-sensitivity analyses. Given lower number of patients and corresponding VTE events in patients receiving the Janssen vaccine, we conducted a separate reduced model analysis with adjustments for age, history of atrial fibrillation, history of VTE, and outpatient versus inpatient status of patients; no significant difference was noted for VTE after Janssen vaccination (aHR: 1.04, 95% CI: 0.77–1.41).

**Table 2:** Adjusted hazard ratio of venous thromboembolism comparing COVID-19 to influenza vaccinations in specified subgroups

Variable	aHR COVID-19 vs. influenza vaccine	95% CI
Age		
<65 y	1.06	0.95–1.18
≥65 y	0.93	0.80–1.07
Sex		
Female	1.07	0.91–1.26
Male	0.91	0.78–1.06
Location		
Outpatient	1.00	0.89–1.12
Inpatient	0.92	0.61–1.39
Type of COVID-19 vaccine		
mRNA (Pfizer and Moderna)	1.01	0.88–1.15
Adenoviral-vector (Janssen)	1.08	0.79–1.47

Abbreviations: aHR, adjusted hazard ratio; CI, confidence interval.

## Discussion

This study is the largest, multicentered study to date to examine the risk of VTE associated with COVID-19 vaccination in comparison to influenza vaccination. The study showed higher rates of VTE in the influenza-vaccinated cohort in unadjusted analysis that could be attributed to more comorbidities in this group compared with the COVID-19 vaccination group. No significant difference in rates of VTE at 90 days were seen between the influenza and COVID-19 vaccinations after multivariable adjustment. Furthermore, additional sensitivity analyses based on age, sex, outpatient versus inpatient status of patients at the time of vaccination, and type of COVID-19 vaccine (mRNA vs. adenoviral vector vaccine) showed no difference in VTE rates between the cohorts.

Safety surveillance of the influenza and the three FDA-approved COVID-19 vaccines through The Vaccine Safety Datalink has not shown an increased risk of DVT and PE. 20 21 22 With vast swaths of populations receiving COVID-19 vaccinations within a concentrated period of time, understanding the associated risk of VTE requires interpreting the observed events in the context of rates of VTE in the absence of exposure to COVID-19 vaccine. The results of the current study are in line with prior studies comparing rates of VTE between vaccinated and unvaccinated populations or during periods of exposure versus nonexposure to COVID-19 vaccine. A large study of 792,010 adult patients vaccinated with any one of the three FDA-approved COVID-19 vaccines in the United States examined the rates of VTE in the 90-day period after versus before vaccination. 13 The aHR after multivariable adjustment for COVID-19 infections, surgeries, or hospitalizations was 1.00 (95% CI: 0.87–1.15) for Pfizer, 1.02 (95% CI: 0.87–1.19) for Moderna, and 0.97 (95% CI: 0.63–1.50) for Janssen. Comparative data on rates of DVT and PE in 884,828 COVID-19 vaccinated versus unvaccinated controls (matched for socio-demographic variables and clinical variables including comorbidities and pregnancy) from Israel showed no significant difference in VTE risk (risk ratio of 0.87 [95% CI: 0.55–1.40] and 0.56 [95% CI: 0.21–1.15] for DVT and PE, respectively]. 14 Another large study from Germany looked at rates of VTE in 326,833 adult patients who received COVID-19 vaccine and a control group of 326,833 individuals matched for age, sex, index month of visiting the health care facility, and diagnoses known to be associated with risk of thromboses within 12 months of index visit. 15 Over an average period of follow-up of 38 days for the vaccinated cohort and 34 days for the nonvaccinated cohort, they reported a nonsignificant incidence rate ratio of 1.06 (95% CI: 0.93–1.22). Similarly, a self-controlled case series from New Zealand of patients aged 12 years and older admitted with any venous or arterial thrombotic events over a 1-year period from February 2021 to 2022 showed an incidence rate ratio of 0.87 (95% CI: 0.76–1.00) within 21 days after Pfizer vaccination compared with no vaccination among 5,127 patients admitted for VTE. 16 Overall, these studies do not suggest an increased risk of VTE when comparing to an unvaccinated control or using a before and after comparison.

Inherent differences between people willing to be vaccinated and unvaccinated individuals can be difficult to measure. Moreover, the thrombogenicity associated with immune stimulation specific to COVID-19 vaccination would be better assessed when evaluated against a comparable immune stimulant. Therefore, the current study compared VTE risk between cohorts of individuals vaccinated against two different vaccines. The results of the current study do not support the findings from a previous study that found higher rates of thrombosis after COVID-19 vaccinations compared with influenza vaccinations. 17 Dr. Vallone and colleagues compared rates of symptomatic thrombosis, both venous and arterial, after COVID-19 (Gam-COVID-Vac or ChAdOx1 nCoV-19 or BBIBP-CorV) or influenza vaccination in outpatients within 30 days postvaccination at a single center in Argentina. Among COVID-19-vaccinated and influenza-vaccinated patients at 30 days, the overall frequency of total thromboembolic events was 0.12% (36/29,918) and 0.06% (15/24,753) respectively (log-rank *p* = 0.02). After adjustment for age, sex, previous thromboembolic event, and major surgery, a significant higher risk of VTE after COVID-19 vaccination remained (aHR: 1.97 [95% CI: 1.08–3.60]). Of note, the increased rate of thrombosis in the COVID-19 cohort was driven by a higher rate of acute coronary syndrome (ACS) alone; there was no statistically significant difference reported between the cohorts in rates of VTE, acute ischemic stroke, or other arterial thrombotic events. There were more patients on antithrombotic medications in the influenza vaccine cohort as compared with the COVID-19 vaccine cohort, which could have led to lower rates of new thrombotic events, including ACS, in the influenza cohort, and this and many other comorbidities were not accounted for in multivariable models. The COVID-19 vaccines used in the Vallone et al study differed from what is approved in the United States and included the Gam-COVID-Vac (Sputnik), ChAdOx1 nCoV-19 (AstraZeneca/Oxford or Covishield), and BBIBP-CorV (Beijing Institute of Biological Products) (Sinopharm) vaccines. These significant differences in the study population, study outcomes, and the type of COVID-19 vaccines administered could explain the difference in the results between the two studies. In addition, though both the prior and current studies used similar means for capturing outcome events, comparatively higher rates of VTE in the two vaccination cohorts in the current study are in part due to a longer period of follow-up of 90 days. Regardless of the differences, the overall low rates of thrombosis observed in both cohorts are reassuring and provide supportive evidence of the low thrombotic risk after COVID-19 vaccination. Importantly, when comparing the outcome of VTE alone, both studies showed no difference between COVID-19 and influenza vaccination cohorts.

A few limitations of our study need acknowledgment. The study being retrospective comes with limitations inherent to this study design including presence of confounding patient-related factors and possible incomplete capture of study outcomes. To address the above, we (1) used multivariable adjustment to account for potential confounders, and conducted additional sensitivity analyses by stratifying the study population based on age, sex, inpatient versus outpatient status at the time of vaccination, and the type of COVID-19 vaccine received, (2) included a comparator arm of post-influenza vaccination patients from a time period prior to the COVID-19 pandemic to enhance the validity of the results within the scope of a retrospective study, and (3) used previously validated NLP algorithms which have shown high accuracy for identification of thrombotic events in upper and lower extremities and PE, instead of relying on diagnosis codes for VTE. Second limitation of the study is that any thrombotic events in unusual or visceral locations would not have been identified by the NLP algorithms which are not designed or validated for identifying such events. This could have caused potential underestimation of VTE events in our study population, but would have affected both influenza and COVID-19 vaccination cohorts comparably. Lastly, since no information was collected on platelet counts or thrombosis in unusual locations, our study outcomes would not be able to identify possible VITT complications that have been seen with Janssen vaccine, though extremely rarely (1:263,000). 23

## Conclusion

In this large cohort of COVID-19-vaccinated patients in the United States, after accounting for baseline differences in demographics and comorbidities, the risk of VTE over an extended 90-day follow-up period was no different than a pre-COVID-19 cohort of influenza-vaccinated patients. Data from this study further demonstrate the safety of COVID-19 vaccines as it relates to the concern for venous thromboembolic events and provides a meaningful frame of reference for patients who receive their annual influenza vaccination.

## Notes

**What Is Known about This Topic?**

- Severe COVID-19 infection is associated with a high risk of venous thromboembolism (VTE).
- There are concerns about risk of VTE associated with COVID-19 vaccination.

**What Does This Paper Add?**

- The risk of VTE following COVID-19 vaccine is low.
- The risk of VTE following COVID-19 vaccine is similar to that with influenza vaccine.
