# Supplementary material for: Comparison of Venous Thromboembolism Outcomes after COVID-19 and Influenza Vaccinations
Source: TH Open. 2023 Dec 4;7(4):e303–8. doi: 10.1055/a-2183-5269 (PMC10695705; doi:10.1055/a-2183-5269)
Supplement: Supplementary file 1 — Supplementary Material [file 10-1055-a-2183-5269-s23080034.pdf]

Supplementary Table S1 Distribution of comorbidities in Charleston comorbidity index

	COVID-19 vaccinated	Influenza vaccinated	*p*-Value
	n (%)	*n* (%)	
AMI	16,864 (1.85)	10,240 (2.31)	<0.001
Cancer	124,650 (13.68)	60,184 (13.60)	0.21
Cancer, metastatic	16,648 (1.83)	11,408 (2.58)	<0.001
CHF	48,109 (5.28)	28,998 (6.55)	<0.001
CTD	41,400 (4.54)	21,249 (4.80)	<0.001
CVA	35,655 (3.91)	22,161 (5.01)	<0.001
Dementia	23,061 (2.53)	13,003 (2.94)	<0.001
Diabetes	111,154 (12.20)	67,281 (15.20)	<0.001
Diabetes with complications	38,430 (4.22)	25,690 (5.80)	<0.001
HIV	25,842 (2.84)	12,995 (2.94)	0.001
Liver	61,143 (6.71)	32,163 (7.27)	<0.001
Liver, severe	7,486 (0.82)	4,166 (0.94)	<0.001
Hemiplegia	6,631 (0.73)	4,050 (0.92)	<0.001
PUD	17,215 (1.89)	9,693 (2.19)	<0.001
Pulmonary disease	139,473 (15.30)	86,933 (19.64)	<0.001
Peripheral vascular disease	42,231 (4.63)	25,268 (5.71)	<0.001
Renal	77,757 (8.53)	46,514 (10.51)	<0.001

Abbreviations: AMI, acute myocardial infarction; CHF, congestive heart failure; CTD, connective tissue disease; CVA, cerebrovascular accident; HIV, human immunodeficiency virus; PUD, peptic ulcer disease.

 © 2023. The Author(s).
